# An electrochemical sensor based on copper-based metal–organic framework-reduced graphene oxide composites for determination of 2,4-dichlorophenol in water†

**DOI:** 10.1039/d0ra06700h

**Published:** 2020-11-19

**Authors:** Manh B. Nguyen, Vu Thi Hong Nhung, Vu Thi Thu, Dau Thi Ngoc Nga, Thuan Nguyen Pham Truong, Hoang Truong Giang, Pham Thi Hai Yen, Pham Hong Phong, Tuan A. Vu, Vu Thi Thu Ha

**Affiliations:** Institute of Chemistry (IoC), Vietnam Academy of Science and Technology (VAST) 18 Hoang Quoc Viet, Cau Giay Hanoi Vietnam Havt@ich.vast.vn; University of Science and Technology of Hanoi (USTH), Vietnam Academy of Science and Technology (VAST) 18 Hoang Quoc Viet, Cau Giay Hanoi Vietnam thuvu.edu86@gmail.com; CY Cergy Paris University, LPPI 5 Mail Gay Lussac F-95000 Cergy France

## Abstract

In the present work, we reported the fabrication of a novel electrochemical sensing platform to detect 2,4-dichlorophenol (2,4-DCP) by using a copper benzene-1,3,5-tricarboxylate–graphene oxide (Cu–BTC/GO) composite. The sensor was prepared by drop-casting Cu–BTC/GO suspension onto the electrode surface followed by electrochemical reduction, leading to the generation of an electrochemically reduced graphene oxide network (ErGO). By combining the large specific area of the Cu–BTC matrix with the electrical percolation from the graphene network, the number of accessible reaction sites was strongly increased, which consequently improved the detection performance. The electrochemical characteristics of the composite were revealed by cyclic voltammetry and electrochemical impedance spectroscopy. For the detection of 2,4-DCP, differential pulse voltammetry was used to emphasize the faradaic reaction related to the oxidation of the analyte. The results displayed a low detection limit (83 × 10^−9^ M) and a linear range from 1.5 × 10^−6^ M to 24 × 10^−6^ M alongside high reproducibility (RSD = 2.5% for eight independent sensors) and good stability. Importantly, the prepared sensors were sufficiently selective against interference from other pollutants in the same electrochemical window. Notably, the presented sensors have already proven their ability in detecting 2,4-DCP in real field samples with high accuracy (recovery range = 97.17–104.15%).

## Abstract 2

In the present work, we reported the fabrication of a novel electrochemical sensing platform to detect 2,4-dichlorophenol (2,4-DCP) by using a copper benzene-1,3,5-tricarboxylate–graphene oxide (Cu–BTC/GO) composite.

## Graphical abstract

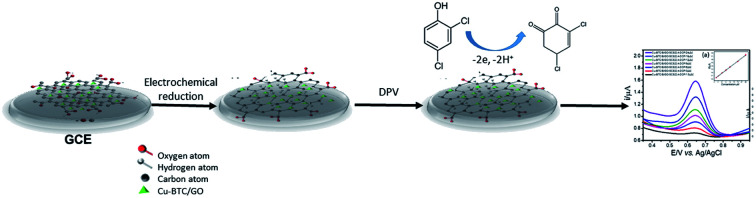

## 1. Introduction

The analysis of phenolic compounds in natural waters and effluents is a strategic subject in environmental control. Phenolic compounds usually coexist in environmental samples and are toxic to animals and aquatic organisms. Both the European Union and US Environmental Protection Agency^1^ consider 2,4-dichlorophenol (2,4-DCP) as a priority pollutant with an admissible concentration in water of 0.5 ng mL^−1^ and it is harmful to human health even at a very low concentration.^2^ Therefore, this compound has received a great interest and it is certainly necessary to develop a simple, fast, sensitive and accurate analytical methods for its detection in environment. For this purpose, many methods have been developed and published, such as gas chromatography,^3^ liquid chromatography,^4^ UV-spectrophotometry,^5^ chemiluminescence^6^ and electrochemical techniques. Among them, the electrochemical methods were more and more interested as they are able to provide low detection limit (LODs), high selectivity and wide linear ranges with no expensive equipment required. Moreover, the equipment is able to perform analysis on-site, therefore, this is very useful for environmental monitoring.

Many applications of the electrochemical detection of 2,4-DCP has been reported using various modified electrodes. J. Zhang *et al.* used molecularly imprinted polymer (MIP) adherenced on GCE^7^ while a ternary composite of diamond, graphene and polyaniline (DGP)^8^ was used as an electrode modifier. With the later modifier, the peak current of 2,4-DCP is visually increased which is explained by the enhancement of electro-active surface of the electrode. Other modifiers have been developed such as β-cyclodextrin functionalized ionic liquid,^9^ enzymatic amplified on graphene membrane,^10^ molybdenum disulfide, ionic liquid and gold/silver nanorods,^11^ metal organic framework Cu_3_(BTC)_2_ carbon paste electrode^12^*etc.*

In the last decade, much effort has been devoted to the integration of Metal–Organic Frameworks (MOFs) based on transition metals onto electrochemical sensing platforms. The focal metals (Zn, Mn, Ni, Cu) in framework acts as active sites to accelerate electrocatalytic reactions whereas organic ligands (1,4-benzene dicarboxylate (BDC), 1,3,5-benzene tricarboxylate (BTC), 4,4′,4′′-benzene-1,3,5-triyl-tribenzoate (BTB)) act as adsorption sites towards target organic molecules.^13^ As a classical MOFs material, Cu–BTC has been widely used in various fields such as gas storage, catalysts and energy materials,^14^ however, its application in electrochemical sensors are still being interested of scientists. Yu Cao *et al.* developed an electrochemical sensor on the hierarchically porous Cu–BTC MOF platform for glyphosate determination.^15^ Ji *et al.* prepare Cu–BTC frameworks as a novel detection platform for sunset yellow and tartrazine.^16^ A Cu–MOF/graphene composite has been used for determination of dopamine and paracetamol,^17^ Cu-based metal–organic framework [Cu_3_(BTC)_2_(H_2_O)_3_]_*n*_ for catechol^18^ and MOF 199-GO-*n*/GCE for catechol and hydroquinone.^19^ Dihydroxylbenzene has been detected in water by using an electrochemical sensor based on Cu–MOF–GN composites.^20^ As Sheying Dong^12^ reports an application of [Cu_3_(BTC)_2_] as a modifier for carbon paste electrode to detect 2,4-DCP and the authors attribute the high sensitivity of the modified electrode to the large surface area, high adsorption capacity and good electron transfer efficiency of the [Cu_3_(BTC)_2_]. Sensitivity and detection limit achieve 0.159 μA μM^−1^ and 0.009 μM respectively in this case. Although MOFs provide a large surface area, high adsorption capacity and good electron transfer efficiency, their disadvantages are known as having low electric conductivity. A simple and efficient strategy to improve the conductivity is to use the mixture of MOFs and some conductive materials, such as carbon materials.^21^ “Graphene and graphene oxide (GO) have been used as substrates for immobilizing and/or conducting the nucleation and growth of MOFs”.^22^ Wang *et al.* reported that graphene oxide (GO) was torn into small fragments to participate in the formation of HKUST-1 composite (HKUST-1 is a copper-based metal–organic framework also known as MOF-199 or Cu–BTC). Their study showed that the composite greatly increased the redox-activity, surface area and electrical conductivity.^23^ Camille *et al.* also demonstrated the synergetic effect between the GO and MOF, including porosity increasing and adsorption enhancement.^24^ The MOF-rGO combination not only facilitate the utilization of the other active materials, but also enhances the mechanical strength and conductivity of the materials synergistically.^25^ In this papers, the Cu–BTC/GO was synthesized by a simple method and used as the electrode material for sensitive detection of 2,4-DCP.

## 2. Experimental

### 2.1. Materials

Benzene-1,3,5-tricarboxylic acid, (H_3_BTC, 98%), CuCl_2_·2H_2_O (97%), NaOH (98%), KH_2_PO_4_, K_2_HPO_4_, C_2_H_5_OH (96%), potassium ferrocyanide trihydrate (K_4_[Fe(CN)_6_]·3H_2_O) and potassium ferricyanide (K_3_[Fe(CN)_6_]) were all purchased from Sigma-Aldrich. All chemicals were used as received without any further treatments.

**Fig. 1:**
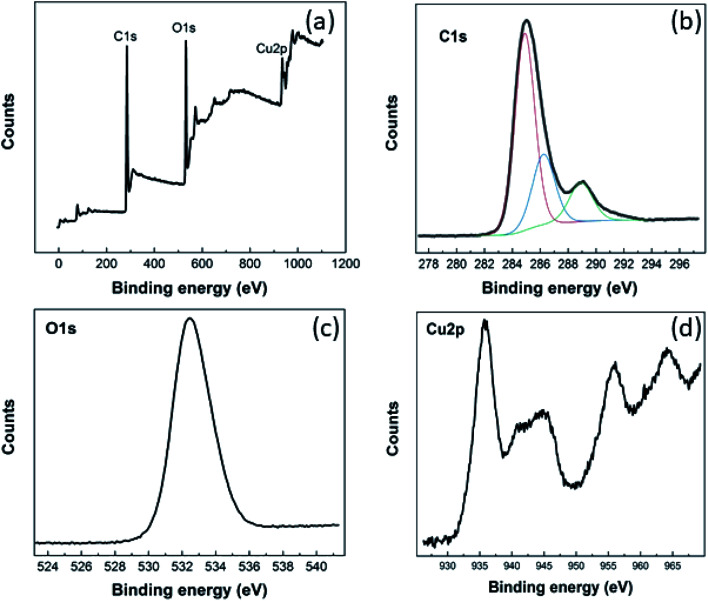
XPS survey (a) and high-resolution C 1s (b), O 1s (c), Cu 2p (d) of Cu–BTC/GO powder.

2,4-Dichlorophenol (2,4-DCP) was purchased from Sigma-Aldrich (structures in Fig. 1S†). Glassy carbon (GCE) (BAS, diameter = 3 mm) was used as current collector. Phosphate buffer solution (PBS) was prepared by mixing 0.1 M KH_2_PO_4_ and 0.1 M K_2_HPO_4_ until a desired pH (in the range from 6.0 to 8.0) was obtained.

### 2.2. Synthesis of nano Cu–BTC/GO composites

Graphene oxide (GO) was synthesized according to modified Hummer method.^26^ Briefly, 1.0 g of graphite flakes were transferred into a mixture of 150 mL concentrated H_2_SO_4_ and H_3_PO_4_ (4 : 1, v/v (%)), stirred at room temperature for 1 h then 6.0 g of KMnO_4_ was added slowly in the solution. The mixture was kept at room temperature for 3 days. Afterward, color of this mixture was turned from dark green to dark brown. The oxidation reaction was quenched using 600 mL ice-cold water and 3 mL H_2_O_2_ (30 wt%). The color of the solution was changed into bright yellow then it was centrifuged and washed with 1 M HCl to remove excess manganese salt. Finally, it was washed with distilled water until neutral pH obtained then dried at 100 °C overnight. The dried compound graphene oxide was labeled as GO.

The synthesis procedure of Cu–BTC/GO is as followed: 2.05 g of CuCl_2_·2H_2_O was dissolved in 97.2 mL of distilled water (solution 1) and 1.68 g of H_3_BTC was added into 23.72 mL of a 1 M NaOH solution (solution 2). Both solutions above were kept under stirring until obtaining clear solutions. The solution (2) was added dropwise to the solution (1) with stirring for 1 h until the mixture turned from green to brown color. Then, 0.8 g of GO was added into the mixture and stirred for 0.5 h at room temperature. Finally, the obtained mixture was poured into a Teflon bottle and heated in a microwave furnace (700 W) at 100 °C for 30 minutes. The product was collected by centrifugation at 8000 rpm for 5 min and washed with distilled water (three times) and finally with ethanol (once). The solid product was turned in brown color after drying in a furnace at 80 °C, overnight.

### 2.3. Characterization of synthesized Cu–BTC/GO

The crystalline phase structure of as-prepared materials was determined over the 2-theta range of 2–50° (D8 ADVANCE, Bruker, Germany) using a Cu K_α1_ copper radiation (*λ* = 0.154 nm) as the X-ray source at a scan rate of 2° min^−1^. FT-IR spectra are recorded on a Bruker TENSOR37 instrument. Scanning electron microscopy images are conducted on a JSM 740, operating at an accelerating voltage of 200 kV. Energy-dispersive X-ray spectroscopy analysis (EDX) was measured on a JED-2300 with gold coating. The Brunauer–Emmett–Teller (BET) surface areas of the samples were evaluated by the N_2_ adsorption isotherm at 77 K using a BET Sorptometer (Automated Sorptometer BET 201-A, USA). TGA data were recorded on the LABSYS evo TG-DTA 1600. X-ray photoelectron spectroscopy (XPS) was measured using a Thermo ESCALAB spectrometer (USA) employing a monochromic Al Kα source at 1486.6 eV. Software used in this case was “Avantage” (purchased from Thermo Fisher Scientific). The deconvolution is performed as followed: the background was generated by using Smart background from the Avantage which is based on the Shirley background with additional condition that the background's values could not exceed the input data at any point in the measured window. The spectrum was fit using the function “Peak fitting” which allows an automatic adjustment of shape parameters (height, width, ratio Gaussian/Lorentzian).

All electrochemical measurements were performed with an AUTOLAB potentiostat PGS302N (Metrohm, Netherland) equipped with a three-electrode configuration, where an Ag/AgCl/Sat. KCl and a Pt wire was used as the reference and counter electrodes, respectively, and modified Cu–BTC/GO electrode was employed as the working electrode. All experiments in this study were performed at room temperature (25 ± 1 °C).

### 2.4. Preparation of Cu–BTC/GO/GCE

Prior to the modification, the GCE surface (3 mm diameter) was polished with 0.5 mm alumina slurries, rinsed thoroughly with double distilled water, sonicated 3 min in acetone and 3 min in water, and dried at room temperature. Three suspensions of Cu–BTC/GO were prepared by dispersing 0.5, 1.0 and 3.0 mg of as-synthetized composite, respectively, in 1 mL of ethanol using ultrasonic stirring for 30 min. A droplet of 5 μL of this dispersion was coated on the well prepared GCE surface and then ethanol was evaporated at room temperature to form Cu–BTC/GO/GCE in about 1 hour. Finally, the modified electrode was thoroughly rinsed with distilled water and placed into the electrochemical cell. Before use, Cu–BTC/GO/GCE was electrochemical reduced by applying a potential of −1.5 V for 300 s in PBS 0.1 M (pH = 7). This electrode was denoted as Cu–BTC/ErGO/GCE.

### 2.5. Optimization of sensor operating conditions

The detection of 2,4-DCP was carried out in aqueous media using differential pulse voltammetry (DPV) method with a differential step of 0.005 V, a sampling time of 0.04 s, a pulse width of 0.08 s and a pulse amplitude of 0.05 V. To improve the effectiveness of sensing performances, some experimental conditions were optimized. Initially, the optimization of the content of Cu–BTC/GO at 0.5, 1.0 and 2.0 mg mL^−1^ solvent used for modification in 0.1 M PBS (pH = 7.0) at 2,4-DCP concentration of 2 ppm with accumulation time of 240 s. The effect pH (6.0, 6.5, 7.0, 7.5 and 8.0) on the electrochemical signals of 2,4-DCP in 0.1 M PBS was also tested. The accumulation conditions of 2,4-DCP onto Cu–BTC/ErGO/GCE were conducted at *i* = 0 (under open-circuit potential) with accumulation time of 60 s, 120 s, 180 s, 240 s, 360 s, 420 s and 480 s. The optimal operating conditions will be selected for sensing performances of as-prepared sensors towards interested analyte in further experiments.

## 3. Results and discussion

### 3.1. Chemical composition and structural behaviors of Cu–BTC/GO

The chemical composition of the Cu–BTC/GO material and valance state of copper were evaluated from XPS spectra (Fig. 1).

**Fig. 2:**
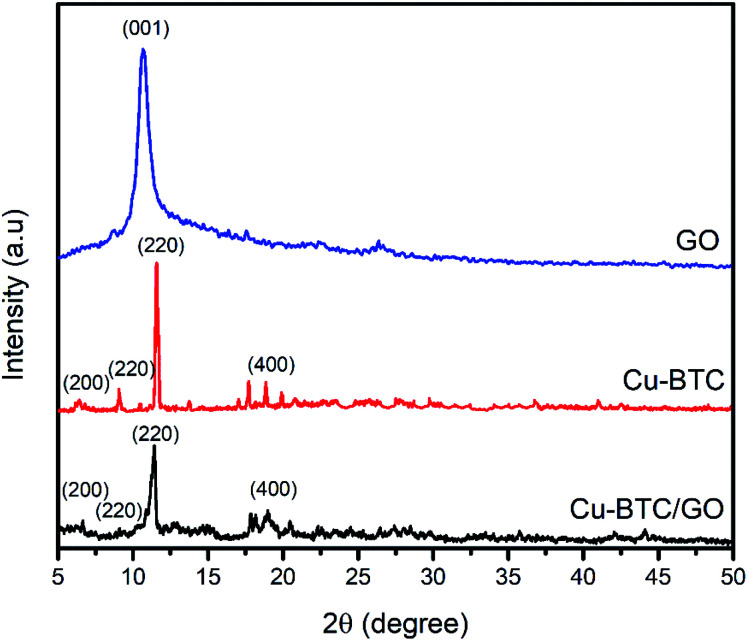
XRD patterns of GO, Cu–BTC and Cu–BTC/GO sample.

As seen from XPS survey spectrum (Fig. 1(a)), the Cu–BTC/GO is mainly composed of carbon (at 74.75%), oxygen (at 21.98%), and copper (at 3.27%) as expected. The high-resolution C 1s spectrum (Fig. 1(b)) shows one main peak at 284.9 eV and three other peaks at higher binding energies. The main peak in C 1s XPS spectrum is probably attributed to C–C and C=C bonds in honeycomb structure of rGO, and in aromatic rings of organic ligands (BTC). The three other peaks arise from the oxygenated functional groups such as C–OH (286.2 eV), C=O (288.9 eV), and O=C–OH (291.1 eV).^27^ The high-resolution O 1 s (Fig. 1(c)) reveals one unique peak at 532.5 eV which is ascribed to oxygen bonding in the crystalline network^28^ as well as residual O bonding in rGO. Cu 2p XPS spectrum (Fig. 1(d)) presents the main peaks of copper (Cu 2p_3/2_ peak at 935.8 eV; Cu 2p_1/2_ peak at 955.8 eV) and the their satellite peaks (Cu 2p_3/2_ satellite at 944.6 eV; Cu 2p_1/2_ satellite at 964.2 eV) which corresponds to copper(ii),^29^ but not copper(i),^30^ copper metal, or copper oxides.^31^ XPS spectra recorded for pure GO was given in the below figures and will be added to ESI (as Fig. 2S†). It can be seen that there is no signal relevant to Cu element in GO sample. In the same time, there are two peaks in O 1s spectrum at 533 eV and 536 eV which are characteristic for GO material.

XRD pattern of Cu–BTC and Cu–BTC/GO represents the peaks at 2*θ* of 6.56°, 9.32°, 11.2°, and 18.92° which are assigned to (200), (220), (222) and (440) reflection planes, respectively.^32^ In the XRD patterns of composites (Fig. 2), there is no characteristic peak of GO, the reason probably lies in the fact that the peak of GO appeared at 2*θ* of 11–12° but this peak is overlapped by the peak at (222) plane located at 2*θ* of 11.2°. The XRD pattern of Cu–BTC/GO showed similar diffraction peaks to bare Cu–BTC,^33^ confirming that the addition of GO does not affect the crystal structure of Cu–BTC.^34^

**Fig. 3:**
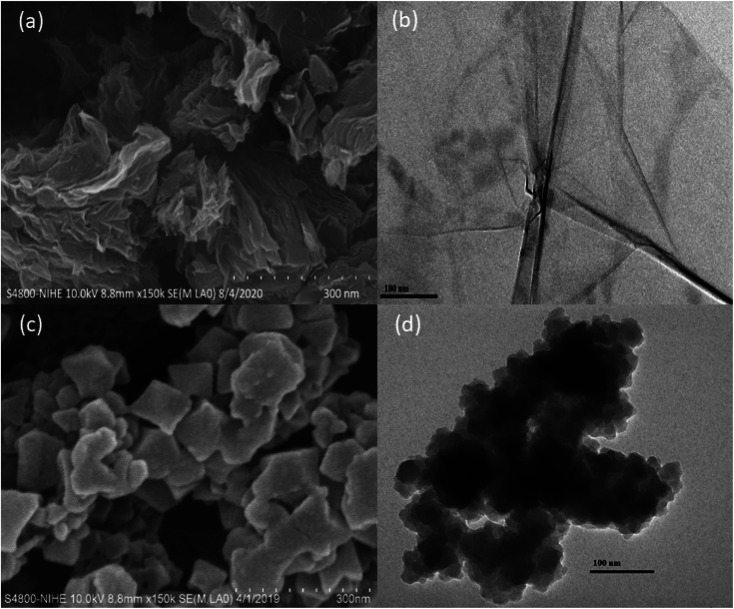
SEM and TEM images of (a and b) GO and (c and d) of Cu–BTC/GO samples.

The FTIR spectra of GO and Cu–BTC/GO is shown in Fig. 3S.† In the spectrum of Cu–BTC/GO, appeared the bands at 1735 cm^−1^ and 1630 cm^−1^ which assigned to the vibration of C=O and carboxylic acid groups, respectively. The bands at 1405 cm^−1^ and 1210 cm^−1^ attributed to the vibration of C–OH, C–O–C and C–O groups, respectively. The band at 3414 cm^−1^ is assigned to the vibration of OH groups that existed in ligands and water. The bands at 1630 cm^−1^, 1570 cm^−1^, 1449 cm^−1^ and 1382 cm^−1^ are attributed to the vibrations of C=O, C–C, C–O groups, respectively.^35^ The bands at 760 cm^−1^, 725 cm^−1^ and 1110 cm^−1^ are attributed to the oscillation of Cu–O bonds.^33^ The absorption peak at 1110 cm^−1^ is characteristic of Cu–BTC with the bond of C–O–Cu.^33^

SEM images (a) and TEM image (b) of Cu–BTC/GO are illustrated in Fig. 3. As observed in Fig. 3, the crystal size of Cu–BTC/GO composites was ranged in 60–80 nm with uniform distribution. The particle size to 60–80 nm when the Cu–BTC crystals are grown and assembled on the GO sheet. Cu–BTC nanoparticles adhere firmly to GO substrate to form a free-standing state composite nanosheet with a sandwich structure.

**Fig. 4:**
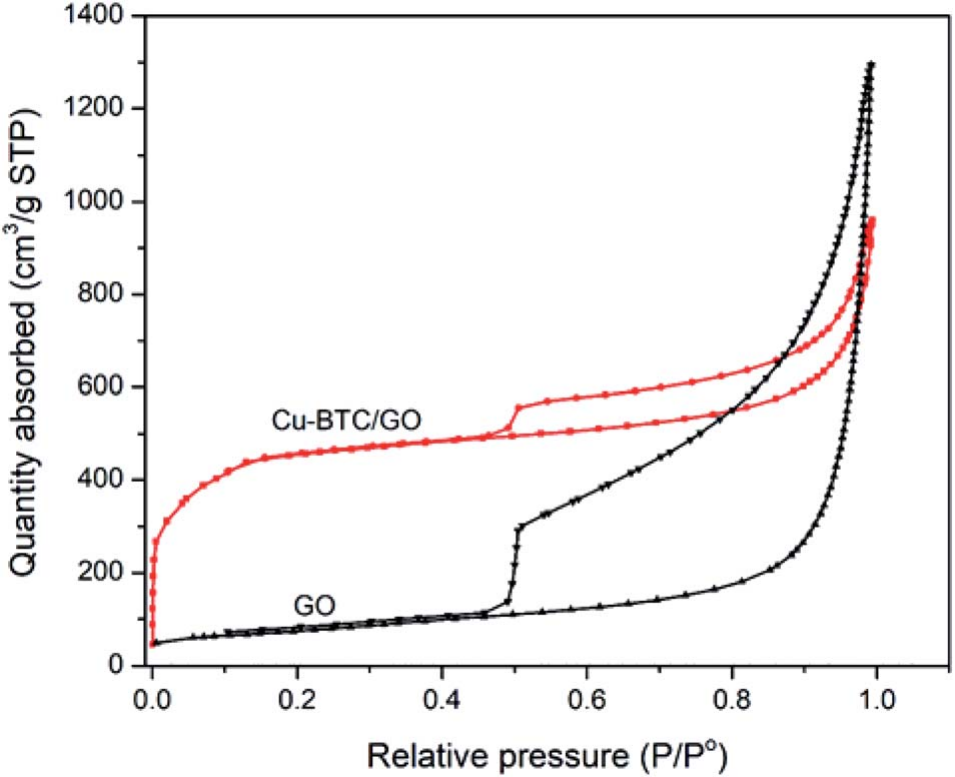
N_2_ adsorption–desorption isotherms of GO and Cu–BTC/GO.

EDX spectrum and EDX mapping image of GO and Cu–BTC/GO were shown in Fig. 4S.† The element content (wt%) of graphene oxide sample with C and O were 76.49 and 23.51%, respectively (Fig. 4S(a)).† As seen in Fig. 4S(b),† the element content (wt%) of C, O and Cu were 56.48, 25.24 and 18.28%, respectively. EDX mapping image of Cu–BTC/GO showed the Cu–BTC particle size of Ca. 50–80 nm with uniform distribution.

### 3.2. N_2_ adsorption–desorption isotherms of Cu–BTC/GO

N_2_ adsorption–desorption isotherms of Cu–BTC/GO samples have a type IV delay curves according to the IUPAC classification. The N_2_ adsorption–desorption isotherms (Fig. 4) at a low partial pressure of 0.4–1.0 appeared the hysteresis loop, which is often observed on mesoporous materials. This behavior is due to the capillary condensation of N_2_ at high partial pressure. The N_2_ adsorption–desorption isotherms of GO showed a larger hysteresis loop as compared to that of Cu–BTC/GO. This can be explained by the fact that space between GO are empty, favoring the capillary condensation of N_2_ while Cu–BTC/GO showed smaller hysteresis loop due to the filling up the Cu–BTC particles within the GO layers. Note that Cu–BTC is a microporous material, there is no the capillary condensation of N_2_ due to the small pore of 0.5–0.8 nm. The surface area, pore volume and pore size of GO were 264 m^2^ g^−1^, 2.02 cm^3^ g^−1^ and 8.95 nm, respectively. The surface area, pore volume and pore size of Cu–BTC/GO were 1591 m^2^ g^−1^, 1.485 cm^3^ g^−1^ and 3.34 nm, respectively.

### 3.3. Thermogravimetric analysis (TGA) and differential thermal analysis (DTA)

**Fig. 5:**
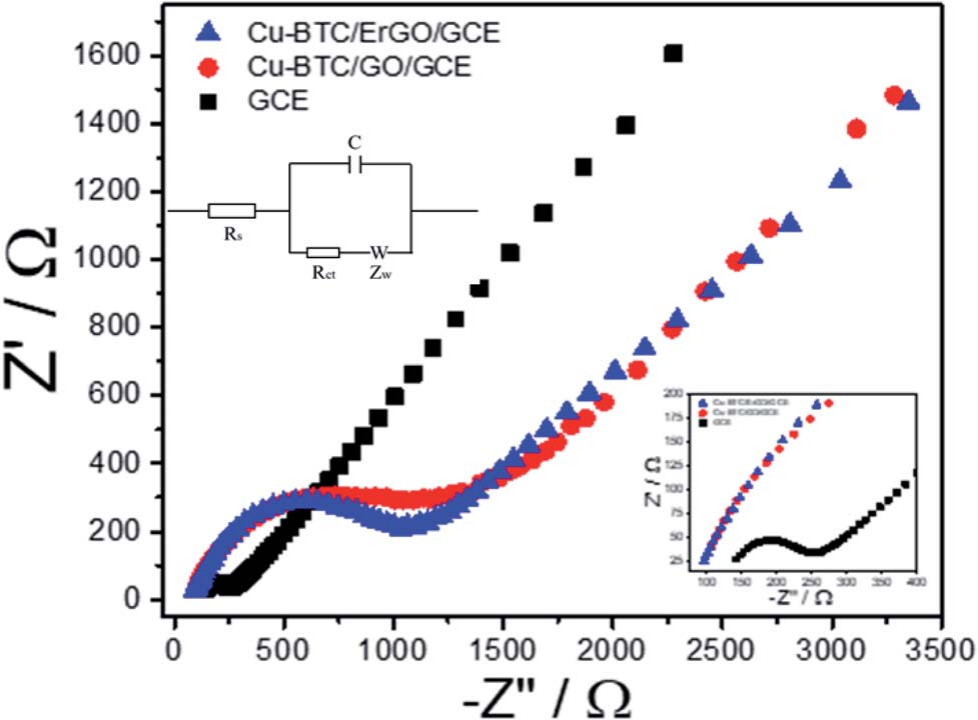
Nyquist diagrams of GCE, Cu–BTC/GO/GCE and Cu–BTC/ErGO/GCE in the solution containing 5 mM Fe(CN)_6_^3−/4−^ and 0.1 M KCl. Parameters as follow: frequency range from 0.01 Hz to 10000 Hz, initiative potential: 0.23 V amplitude: 10 mV and quiet time of 5 s. Its equivalent circuit is in inset.

The thermal stability of Cu–BTC/GO composite with respect to temperature was studied by TGA (Fig. 5S†). As seen Fig. 5S† the initial weight loss of 24.03% at 100 °C can be attributed to loss of moisture which continues up to 150 °C. The weight loss of 55.09% from 250 to 550 °C is may be due to decomposition of organic benzene tricarboxylate linker and collapsing of MOF structure. From TGA diagram, an intense peak at 360.53 °C appeared. This indicated the exothermal process due to the burning Cu–BTC which caused the collapsing Cu–BTC structure.

### 3.4. Electrochemical performance of Cu–BTC/ErGO/GCE

**Fig. 6:**
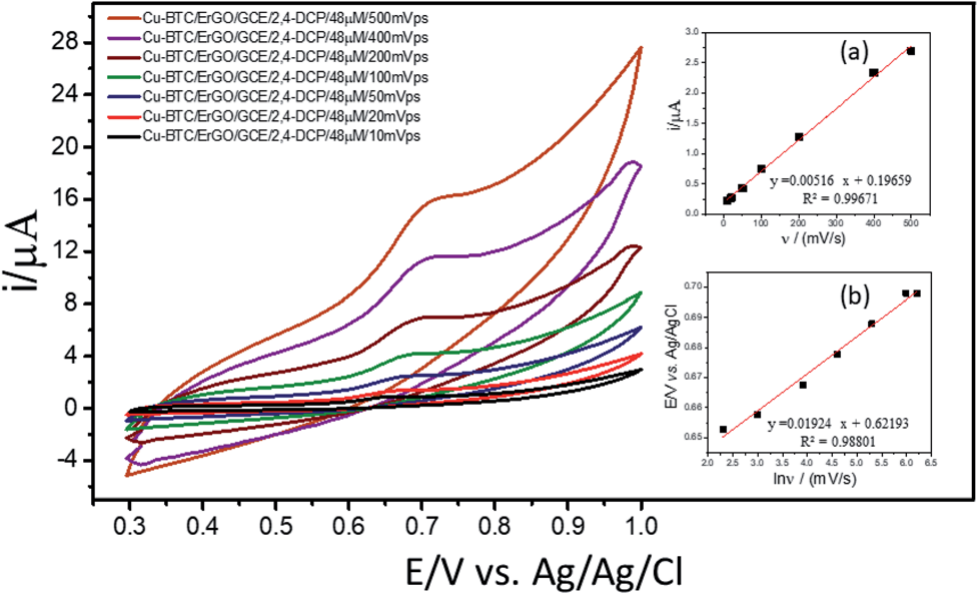
CVs of 2,4-DCP 48 μM in PBS pH 7 recorded on Cu–BTC/ErGO/GCE at different scan rates (*ν*): 10, 20, 50, 100, 200, 400 and 500 mV s^−1^. Inset: relationship between anodic peak currents and scan rate (a); peak position and scan rate (b).

Before using Cu–BTC/ErGO as a modifier of detection of 2,4-DP, DPVs of 12 μM of 2,4–DCP on different electrodes: bare GCE, GO/GCE, ErGO/GCE, Cu–BTC/GO/GCE and Cu–BTC/ErGO/GCE were recorded in PBS pH = 7 (Fig. 6S†). After baseline subtraction, oxidation peaks of 12 μM of 2,4-DCP were observed on all tested materials. Four smaller peaks at 0.59 V, 0.66 V, 0.72 V and 0.81 V received from GCE, Cu–BTC/GO/GCE, Er/GO/GCE and GO/GCE respectively. The highest peak at about 0.65 V assigned to 2,4-DCP oxidation on Cu–BTC/ErGO/GCE. Currents measured in this case are about 1.46, 1.42, 1.65 and 3.8 times higher than that using GCE, Cu–BTC/GO/GCE, ErGO/GCE and GO/GCE respectively. Therefore, Cu–BTC/ErGO was selected for further investigation.

Electrochemical behavior of Cu–BTC/GO/GCE before and after reduction were tested with electrochemical impedance spectroscopy (EIS) and cyclic voltammetry (CVs) using Fe(CN)_6_^3−/4−^couple as the electrochemical redox probes. It is known that the semicircle at higher frequencies is associated with the charge transfer resistance (*R*_ct_) whereas the linear part at lower frequencies is related to the diffusion process. Fig. 5 shows the impedance spectra of bare GCE, Cu–BTC/GO/GCE and Cu–BTC/ErGO/GCE in solution containing 5 mM Fe(CN)_6_^3−/4−^ and 0.1 M KCl.

**Fig. 7:**
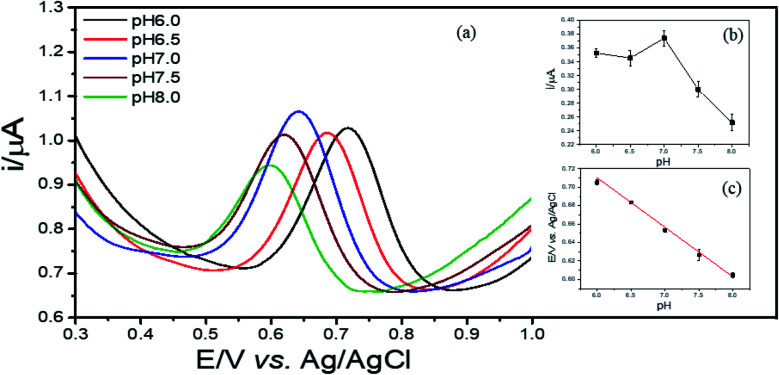
DPVs of 2,4-DCP on Cu–BTC/ErGO/GCE in PBS pH 7 with different pH: 6.0; 6.5; 7.0; 7.5; 8.0 (a), currents varied by pH (b) and relationship between potentials and pH (c).

In the Nyquist diagram (Fig. 5), the bare GCE exhibited a relatively low electron transfer resistance (*R*_ct_ = 146.3 Ω) with a small semicircle (refer to in inset for further detail). After the deposition of Cu–BTC/GO, the electron transfer rate dramatically dropped, which resulted from an increase of the charge transfer resistance (*R*_ct_ = 1407.7 Ω) due to the low conductivity of Cu–BTC/GO. Once the Cu–BTC/GO is electrochemically reduced to Cu–BTC/ErGO, the resulted *R*_ct_ decreases (*R*_ct_ = 1079.7 Ω). Consequently, electrical conductivity of Cu–BTC/ErGO attached on GCE was improved. All the results further demonstrate that the modified electrode is successfully fabricated. This result is in a good agreement with CV tests (Fig. 7S†). Accordingly, the electron transfer properties were evaluated in presence of an inner sphere redox probe (*i.e.* Fe(CN)_6_^3−^). From electrochemical standpoint, the electron transfer in inner sphere systems requires strong interaction between the redox probe and the electrode surface. While bare GCE depicted a well – defined reversible redox system with a peak-to-peak separation (Δ*E*_p_) of 60 mV, the Cu–BTC/GO/GCE exhibits extremely low electron transfer rate with a full blocking effect, resulted from the absence of faradaic signal of Fe(CN)_6_^3−/4-^ in the studied electrochemical window (Δ*E*_p_ > 1.5 V). After reduction to Cu–BTC/ErGO/GCE, the resulted electrode displays intermediate electron transfer rate (Δ*E*_p_ = 200 mV) highlighting an increase of electrical conductivity compared to the non-reduced composite (Fig. 7S†).

#### 3.4.1 Effects of scan rate (*ν*)

**Fig. 8:**
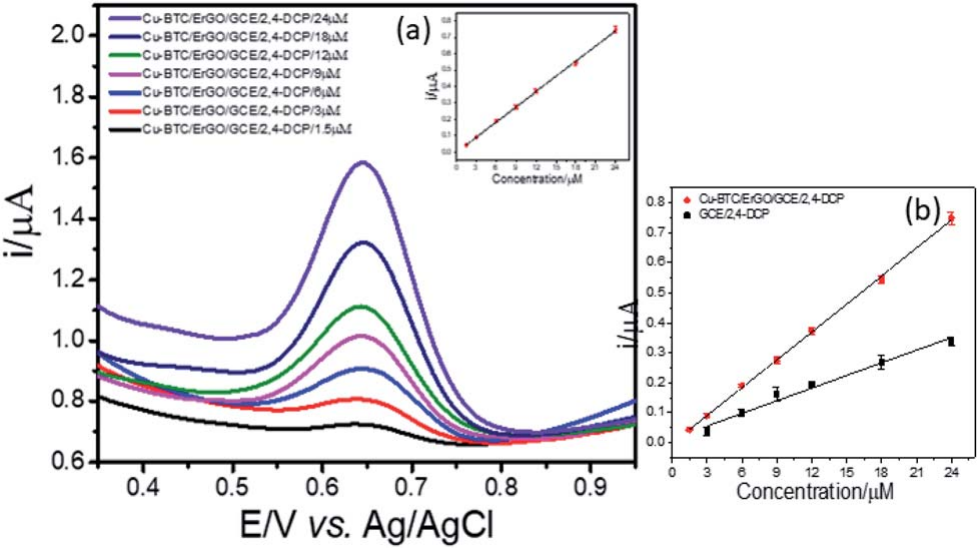
Voltammograms acquired from 2,4-DCP in the concentration range from 1.5 to 24 μM (a) and the variations of peak intensities according to the concentration change using both modified and bare GCE (b).

The electroactive surface area (ECSA) of the electrodes were also tested by scanning CV curves at different scan rates in the range from 10–500 mV s^−1^. A bare GCE was used for comparison. A reduction peak can be observed at about 0.24 V on the CV curves of the electrodes in K_3_[Fe(CN)_6_] solution. This peak corresponds to the reduction of Fe(iii) → Fe(ii). The oxidation peak appears on the backward curves at about 0.09 V, corresponding to Fe(ii) → Fe(iii) oxidation (Fig. 8S†). Both anodic and cathodic peak heights are proportional to the square root of the scanning rate (*i*_p,a_ = 2.2715 sqrt (*ν*) + 7.54 with *R*^2^ = 0.9279). These peak heights were used for further calculations of effective surface areas. At 100 mV s^−1^, the ECSA was estimated (from Randle–Sevcik equation) to be 0.0568 and 0.0265 cm^2^ for bare and modified electrodes, respectively. Observed peaks appeared in Fig. 8S† at 0.711 V and 0.689 V, which peak heights are proportional to scan rate as well. It was suggested that the second redox couple is related to the unexpected formation of iron oxide particles.

For investigation of kinetics of electrochemical oxidation of 2,4-DCP on Cu–BTC/ErGO/GCE, the effect of scan rate on electrochemical signals was recorded with 10, 20, 50, 100, 200, 400 and 500 mV s^−1^ (Fig. 6) in PBS at pH 7. As seen in Fig. 6 (inset (a)), oxidation peak currents increased linearly with the increase of scan rate with the regression equation *i*_p,a_ = 0.0052 × *ν* + 0.1966 (*R*^2^ = 0.9967), which suggests an adsorption controlled kinetic process on the modified electrode surface Cu–BTC/ErGO/GCE.^9,11,12^ As seen in Fig. 6, the oxidation peak potential (*E*_p,a_) slightly shifted to more positive potentials with the increasing of the scan rate. The dependence between *E*_p,a_ and the ln(*v*) was shown in the inset of Fig. 6(b) in linear regression equation of *E*_p,a_(*V*) = 0.0192 ln(*v*) + 0.6219 (*R*^2^ = 0.9880).

As for an adsorption-controlled and totally irreversible electrode process, the relationship between the potential (*E*_p,a_) and the natural logarithm of scan rate (ln(*v*)) could be expressed as follows by Laviron:^36^
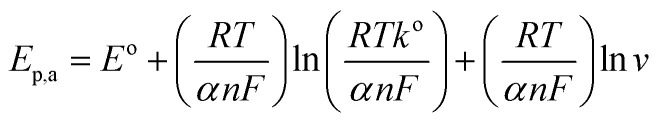
where *v* is the scan rate, *n* is the number of electron transferred, *α* is the electron transfer coefficient, *k*^o^ is standard rate constant of the reaction, and *R*, *F* and *T* are gas constant, faraday constant and absolute temperature, respectively. As the slope of the plot of *E*_p,a_*versus* ln(*v*) (equal to *RT*/*αnF*) is 0.0192, the value of *αn* was about 1.3. Also from Laviron,^36^*α* for an irreversible electrode process is assumed to be 0.5, therefore, the number of electron transferred (*n*) for electro-oxidation of 2,4-DCP is around 2.^9,11,12,37^

### 3.5. Optimization of the electrochemical experiment parameters

#### 3.5.1 Influence of Cu–BTC/GO content

Influence of Cu–BTC/GO content used for modification was also tested from 0.5 mg mL^−1^, 1.0 mg mL^−1^, 2.0 mg mL^−1^. The signals of 2,4-DCP (Fig. 9S†) 12 μM in PBS 0.1 M, pH 7, from 0.2 V to 1.4 V, accumulation time 240 s show that at 1 mg mL^−1^, the curve formed a symmetrical line with the highest current. This content was used for further electrode modifications.

#### 3.5.2 Effect of pH

As the electrochemical oxidation of 2,4-DCP followed a proton coupled electron transfer (PCET) mechanism, *i.e.* 2,4-DCP ⇌ oxidized product + ne^−^ + nH^+^ (*n* = 1 or 2), the reaction rate is directly depending on the electron flux and the concentration of proton in solution.^10,12^ Consequently, the pH becomes one of the key parameters impacting the performance of the presented sensors. DPVs of 2,4-DCP (*C* = 12 μM) were obtained by sweeping the potential from 0.2 V–1.4 V in PBS buffer with pH ranged from 6.0–8.0 at interval 0.5. As shown in the Fig. 7a, the peak potential is negatively shifted as the pH of the solution increases. By plotting the variation of peak potential in function of pH values, a linear relationship is obtained, *i.e. E*_p, a_ = −0.0547 pH + 1.0369, *R*^2^ = 0.9924. Based on this equation and the number of electrons transferred (about 2, above), the electrochemical oxidation of 2,4-DCP at Cu–BTC/ErGO/GCE is a two-electron and two-proton process, which is in good agreement with previous publications.^9,11,12,37^

#### 3.5.3 Effects of accumulation time

The accumulation conditions are essential to improve electrochemical signals recorded during adsorption-controlled electrochemical reactions such as electro-oxidation of 2,4-DCP. Here, the DPV voltammograms from 0.2 V–1.4 V of interested analyte at 3 μM were recorded at different accumulation time (60–480 s) with interval of 60 s in PBS electrolyte (pH 7.0). Bare GCE was also used for comparison with modified electrode (Fig. 10S†). As indicating in this Fig. 9S,† the saturation of 2,4-DCP on both modified and unmodified electrodes was reached at an accumulation time of 240 s. Two other concentrations of 2,4-DCP (6 μM and 12 μM) in a similar condition were investigated and 240 s of adsorption time is observed as optimized duration to achieve maximum electrochemical signals (Fig. 11S†).

### 3.6. Detection of 2,4-DCP using Cu–BTC/ErGO/GCE

#### 3.6.1 Calibration curve

Fig. 8 shows voltammograms of all the samples measured using Cu–BTC/ErGO/GCE under optimized conditions. 2,4-DCP peak heights increased with increase in concentrations with the regression equation of *i* (μA) = 0.0310. *C* (μM) – 0.003 and relative standard deviation (RSD) is 3.52% (Fig. 8(a)). Even the electron transfer at modified electrode was not as good as bare one (section 3.2), the good adsorption of 2,4-DCP into porous structure of Cu–BTC/GO material must be the reason for the enhancement in current responses. For each concentration, the experiment was repeated three times using three separately prepared Cu–BTC/ErGO/GCE.

Next, LOD was calculated based on the response curve in the figure (
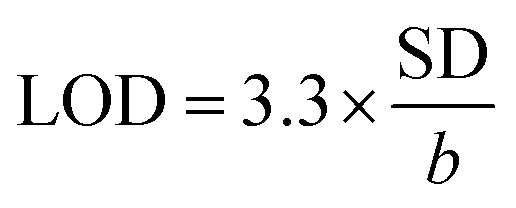
; SD: standard deviation of ordinate intercept, *b*: slope of regression line). The LOD were 0.083 μM for detection of 2,4-DCP in PBS (pH 7). Although the achieved LOD is not best in comparison with those reported in the previous studies (Table 1), it is clearly acceptable and in a top-level group.

**Table 1:** Comparison of sensing performances of electrochemical sensors for detection of 2,4-DCP^a^

Electrode configuration	Method	Linear range (μM)	LOD (μM)	Ref.
AuNPs	UV-visible spectra	0.735–147	1 × 10^−2^	6
Microsomal cytochrome P450-3A4 (CYP3A4) nanobiosensor	SWV	0–1000	3.16 × 10^−4^	7
Diamond, graphene, and polyaniline/GCE	SWV	5–80	0.25	8
β-Cyclodextrin functionalized ionic liquid modified chemical sensor	CV	4–100	1.2	9
Enzymatic amplified on graphene membrane	Amperometry	1.0 × 10^−2^–13	5.0 × 10^−3^	10
MoS_2_–IL–Au/Ag NR/GCE (molybdenum disulfide, ionic liquid and gold/silver nanorods)	DPV	0.01–50	2.6 × 10^−3^	11
Cu_3_(BTC)_2_/CPE modified carbon paste electrode	DPV	0.04–1.0	9 × 10^−3^	12
CPA/GO-OXSWCNTs/GCE	DPV	(0.05–1.2) × 10^−3^	4.2 × 10^−3^	37
PEY/MWNT-OH/GCE	DPV	0.005–0.1, 0.2–40.0	1.5 × 10^−3^	38
Tyrosinase/MWNTs/PDDA/GCE	Amperometry	2–100	0.66	39
Nafion/PSS-GN-CTAB/GCE	LSV	0.01–2.0	2 × 10^−3^	40
PVP/ZnSe-CTAB/GCE	DPV	0.006–9.0	2 × 10^−3^	41
Cu–BTC/ErGO/GCE	DPV	1.5–24	0.083	This work

a AuNPs = gold nano particles, CPE = carbon paste electrode, NPs = nanoparticles, ILs: ionic liquids, NRs = nanorods, BTC = benzenetricarboxylic, GO = graphene oxide, ErGO = electrochemical reduced graphene oxide, GCE = glassy carbon electrode; SWV = square wave voltammetry, DPV = differential pulse voltammetry, CV = cyclic voltammetry, LSV = linear sweep voltammetry, OXSWCNTs = oxidized carbon single wall carbon nanotubes, PEY = poly(eosin Y), MWNT = multi-walled carbon nanotubes, PDDA = polydiallyldimethylammonium chloride, PSS = poly(sodium-4-styrenesulfonate), GN = graphene, CTAB = cetyltrimethylammonium bromide, PVP = polyvinylpyrrolidone.

#### 3.6.2 Reproducibility, repeatability and durability

The reproducibility was tested and the error bars in the response curve correspond to the standard deviations of peak intensities acquired using the eight separate sensors (Fig. 12S†). The magnitudes of error bars are quite small and the average relative standard deviations (RSDs) in the measurements of 2,4-DCP are 2.55%, thereby confirming the reproducible formation of Cu–BTC/GO on GCE. The repeatability was also evaluated by consecutively measuring a sample containing 12 μM 2,4-DCP five times using one sensor. No decreases of peak intensities were observed during the scans, and resulting RSDs of 2,4-DCP peak intensities were 2.03%, respectively (Fig. 13S†). The result confirms the superior surface stability of Cu–BTC/ErGO/GCE.

The durability of the prepared electrode was tested in the presence of 12 μM 2,4-DCP and retained a response of 96.96% and 93.85% after 1 and 2 weeks respectively (Fig. 14S†). It is worth noting that the sensing electrodes were stored in air without any specific protection.

#### 3.6.3 Interference study

Finally, the selectivity of measurements was evaluated by observing the electrochemical signals of 2,4-DCP samples in presence of Hg^2+^/Pb^2+^/Mg^2+^/As^3+^ or 4-nitrophenol, bisphenol A, hydroquinone and dopamine as interferants. The concentrations of interferants were designedly fixed at 60 μM which is five times greater than 2,4-DCP concentration. As displayed in the Fig. 15S,† there are no significant changes in current intensity (only less than 10% peak current of 2,4-DCP decreased with presence of bisphenol A, hydroquinone and dopamine), suggesting the absence of interference effect due to other compounds.

#### 3.6.4 Real sample analysis (lake water)

Finally, Cu–BTC/ErGO/GCE was further used to determine 2,4-DCP concentrations in real samples collected from West Lake (Hanoi, Vietnam) according to Standard Methods for Examination of Water and Waste Water (SMEWW). The samples were directly analyzed without any further treatment. Table 2 shows the determined 2,4-DCP concentrations spiked in the lake water using Cu–BTC/ErGO/GCE and the calculated recoveries (Fig. 16S†).

**Table 2:** Determined 2,4-DCP concentrations spiked in lake water using Cu–BTC/ErGO/GCE. The recovery in each case is also shown

Sample	24 DCP (μM)		
	Added	Found	Recovery (%)
Lake water	3.00	3.13 ± 0.47	104.15
	4.50	4.43 ± 0.10	99.48
	6.00	5.83 ± 0.36	97.17

The concentration determinations using Cu–BTC/ErGO/GCE were accurate with a recovery range of 99.48–104.15%. Therefore, the detection performance of Cu–BTC/ErGO/GCE was acceptable for this purpose.

## 4. Conclusion

In the present work, a Cu–BTC/GO material was prepared using hydrothermal approach and then utilized as electrode modifier for electrochemical determination of 2,4-DCP in aqueous solutions. The as-synthesized material shows nanoscale porous structures with surface area, pore volume and pore size of Cu–BTC/GO were 1591 m^2^ g^−1^, 1.485 cm^3^ g^−1^ and 3.34 nm, respectively. Developed sensors by modifying the electrode surface with such a porous material on GCE followed by electrochemical reduction was used for detection of 2,4-DCP with detection limits of 0.083 μM and linear range from 1.5–24 μM. Sensor-to-sensor reproducibility was estimated by measuring eight separate prepared Cu–BTC/ErGO/GCE sensors and RSDs of the peak intensities of 2,4-DCP were 2.55%, thereby indicating the superior sensor-to-sensor reproducibility. The repeatability was also evaluated by consecutively measuring a sample containing 12 μM 2,4-DCP five times using one sensor and RSDs of 2,4-DCP peak intensities were 2.03%. Real lake water samples were analyzed, the determined concentrations were in good recoveries, which ranged from 97.17.2–104.15%. This is a valuable merit when considering practical field application of the sensor. Therefore, a small and portable analytical device embedded with an Cu–BTC/ErGO/GCE sensor as a promising component is under development for on-site measurement of 2,4-DCP concentrations in water samples.

## Conflicts of interest

There are no conflicts to declare.

## Supplementary Material

RA-010-D0RA06700H-s001
