# Supplementary material for: An electrochemical sensor based on copper-based metal–organic framework-reduced graphene oxide composites for determination of 2,4-dichlorophenol in water
Source: RSC Adv. 2020 Nov 19;10(69):42212–20. doi: 10.1039/d0ra06700h (PMC9057864; doi:10.1039/d0ra06700h)
Supplement: RA-010-D0RA06700H-s001 [file RA-010-D0RA06700H-s001.pdf]

Electronic Supplementary Material (ESI) for RSC Advances.
This journal is © The Royal Society of Chemistry 2020

An Electrochemical Sensor Based on Copper-Based Metal-Organic Frameworks- reduced Graphene Oxide composites for Determination of 2,4 – Dichlorophenol in Water

(Supplementary)

Manh B. Nguyen[1+], Vu Thi Hong Nhung[1+], Vu Thi Thu[2*], Dau Thi Ngoc Nga[2], Thuan Nguyen Pham Truong[3], Hoang Truong Giang[2], Giang H. Le[1], Pham Thi Hai Yen[1], Pham Hong Phong[1], Tuan A. Vu[1] and Vu Thi Thu Ha[1,2*]

[1]Institute of Chemistry (IoC), Vietnam Academy of Science and Technology (VAST), 18 Hoang Quoc Viet, Cau Giay, Hanoi, Viet Nam.

[2]University of Science and Technology of Hanoi (USTH), Vietnam Academy of Science and Technology (VAST), 18 Hoang Quoc Viet, Cau Giay, Hanoi, Viet Nam.

[3]CY Cergy Paris University, LPPI, 5 mail Gay Lussac, F-95000, Cergy, France.

[+]These authors provided equal contribution to this work.

*Corresponding author: Vu Thi Thu Ha, Vu Thi Thu

Email: Havt@ich.vast.vn; thuvu.edu86@gmail.com.

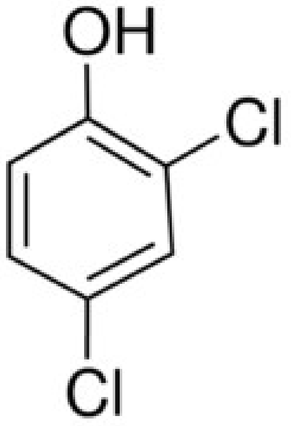

Fig. 1S: Structures of studied 2,4- dichlorophenol

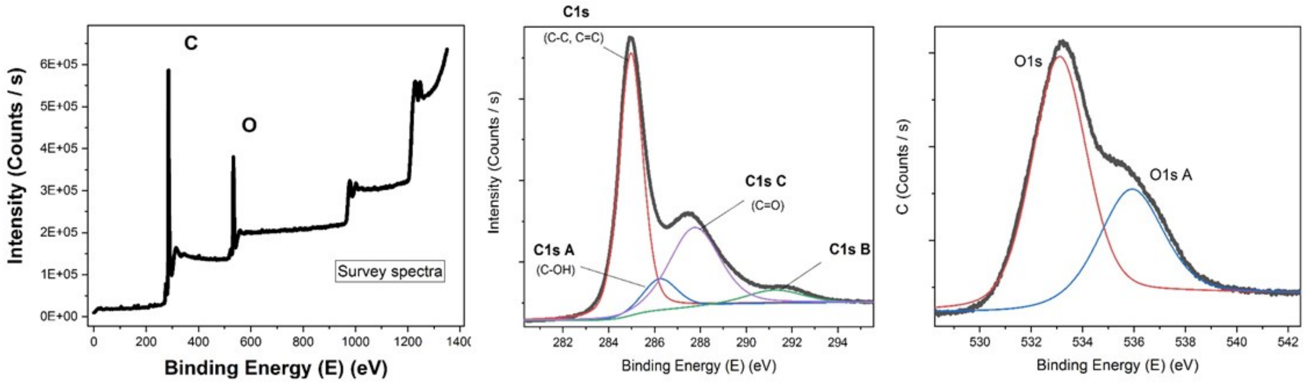

Figure 2S: XPS spectra recorded for pure GO

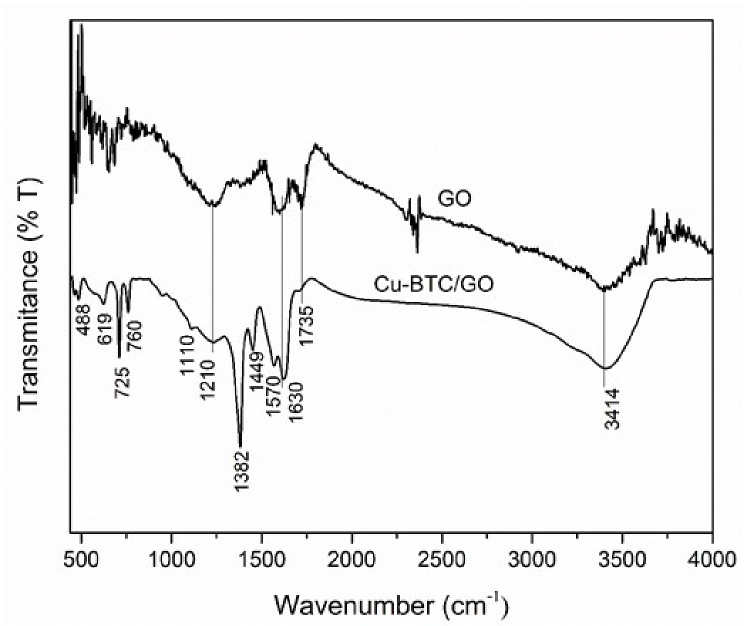

Figure 3S: FTIR spectrum of GO and Cu-BTC/GO

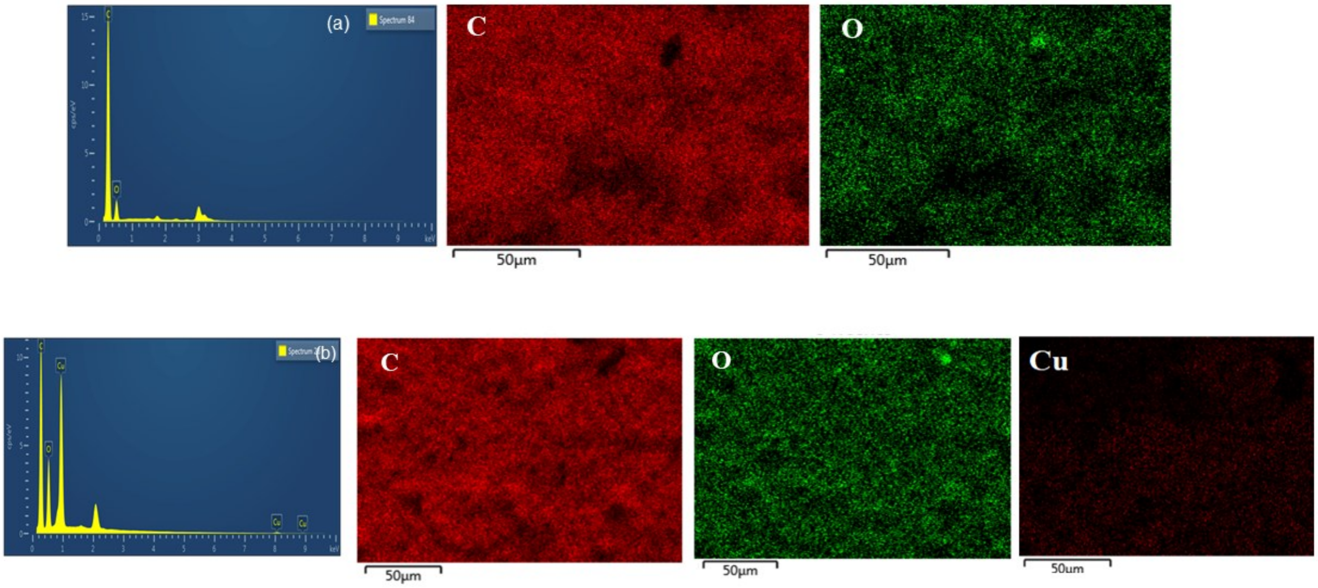

Figure 4S: EDX spectrum and EDX mapping image of GO (a) and of Cu-BTC/GO (b) samples

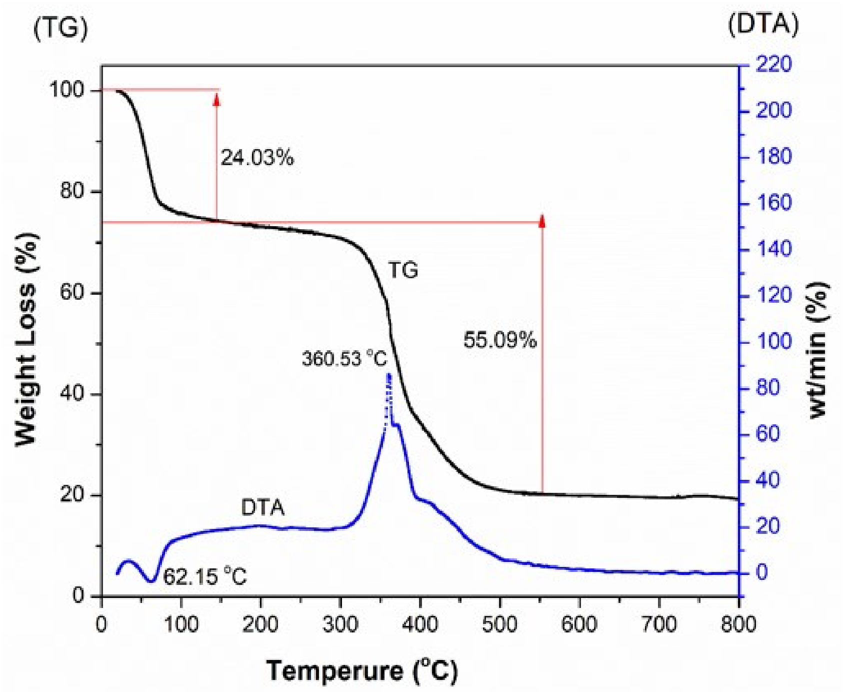

Figure 5S: TGA and DTA diagrams of Cu-BTC/GO sample

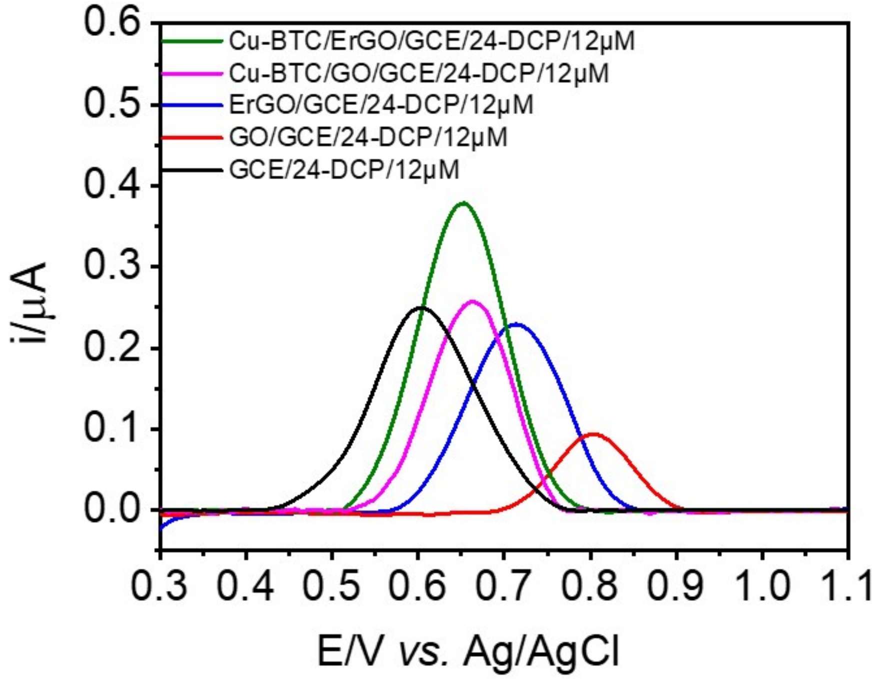

Figure 6S: DPVs of 12µM of 2,4-DCP using different electrodes: bare GCE, GO/GCE, ErGO/GCE, Cu-BTC/GO/GCE and Cu-BTC/ErGO/GCE recorded in PBS, pH = 7 after baseline substraction

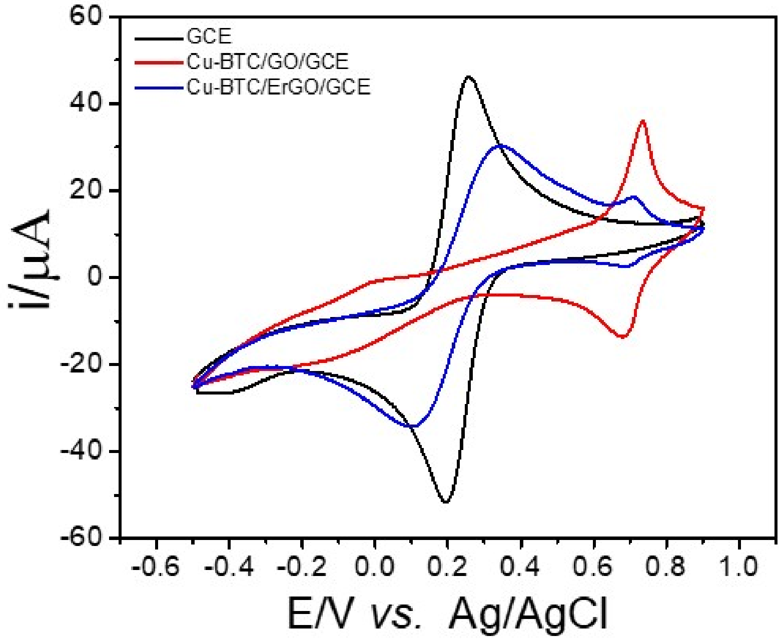

Figure 7S: CVs of bare GCE, Cu-BTC/GO/GCE and Cu-BTC/ErGO/GCE from - 0.5V - 0.9V in $K_3Fe(CN)_6$ 5mM/ PBS 0.1M, pH 7

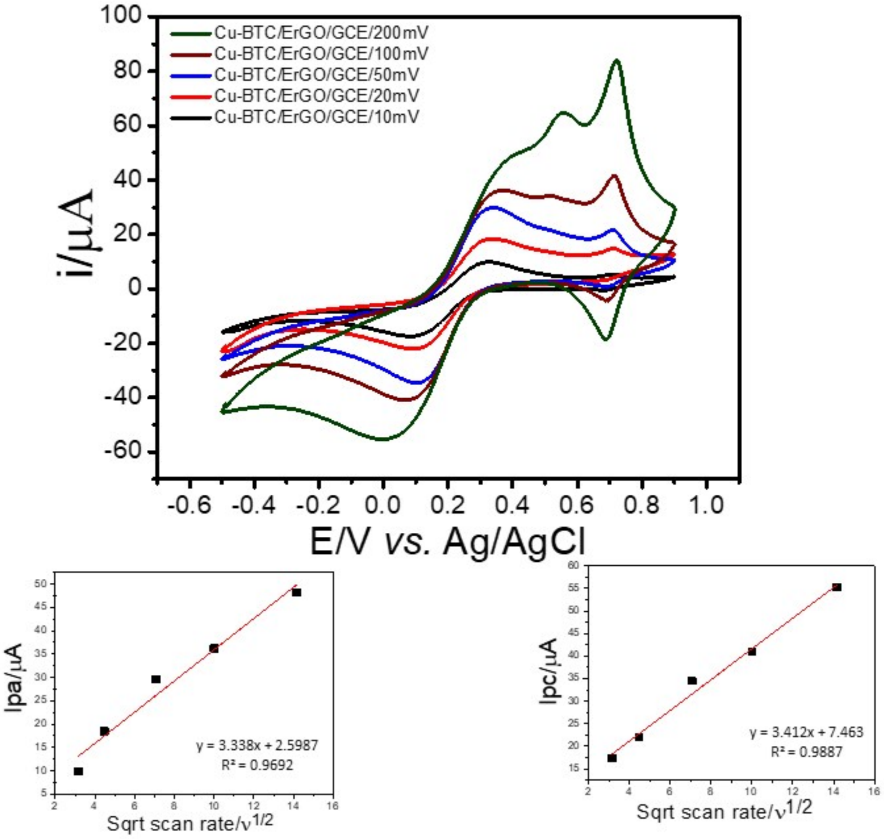

Randle – Sevick equation:

$$I_p = (2.69 \times 10^5) n^{3/2} A C D^{1/2} v^{1/2}$$

$$A \text{ is the active surface area } (ECSA)(cm^2)$$

$$D \text{ is the diffusion coefficient of } [K_3Fe(CN)_6]\ (6.605 \times 10^{-6}\ cm^2 s^{-1})$$

$$n = 1 \text{ is the number of transferred electrons for } [Fe(CN)_6]^{3-/4-} \text{ redox couple}$$

$$C \text{ is the bulk concentration of } [K_3Fe(CN)_6]\ (5\ mM)$$

Figure 8S: CVs of Cu-BTC/ErGO/GCE in 5mM $K_3Fe(CN)_6/K_4Fe(CN)_6$ + 0.1M KCl at different scan rates and calculation of electroactive surface area (ECSA)

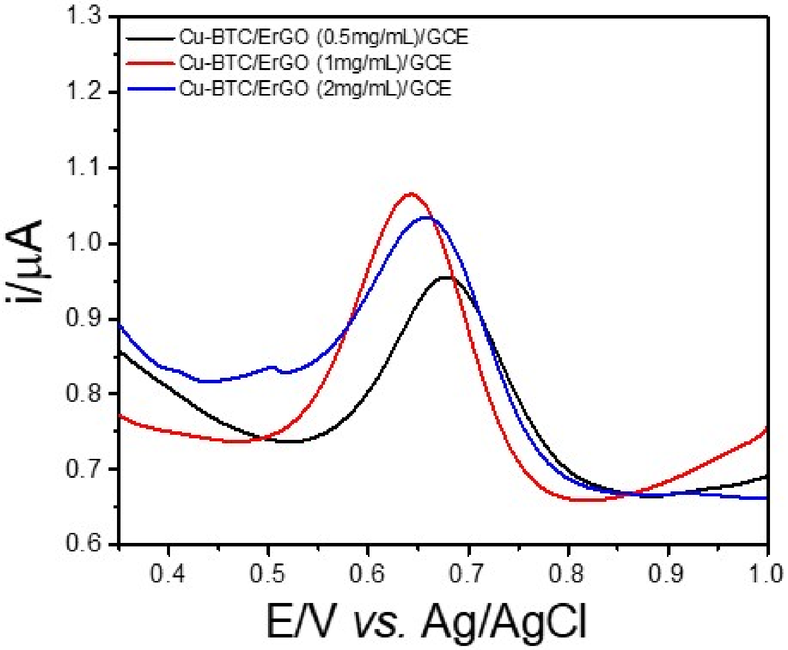

Figure 9S: Influence of content of Cu-BTC/ErGO/GCE used for modification on electrochemical signals of 2,4-DCP

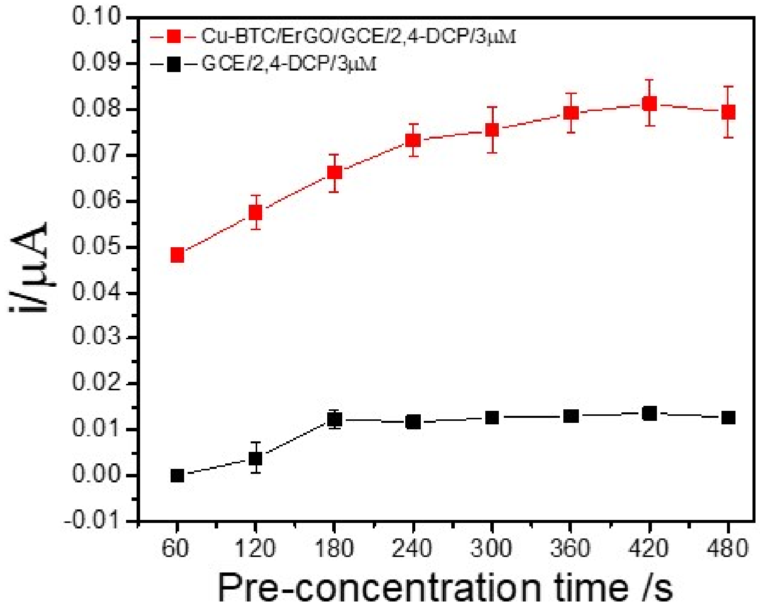

Figure 10S: Influence of accumulation time (60- 480 s) on DPV signals recorded in PBS solution (pH 7.0) containing of 2,4-DCP at concentration of 3 μM on Cu-BTC/ErGO/GCE and bare GCE

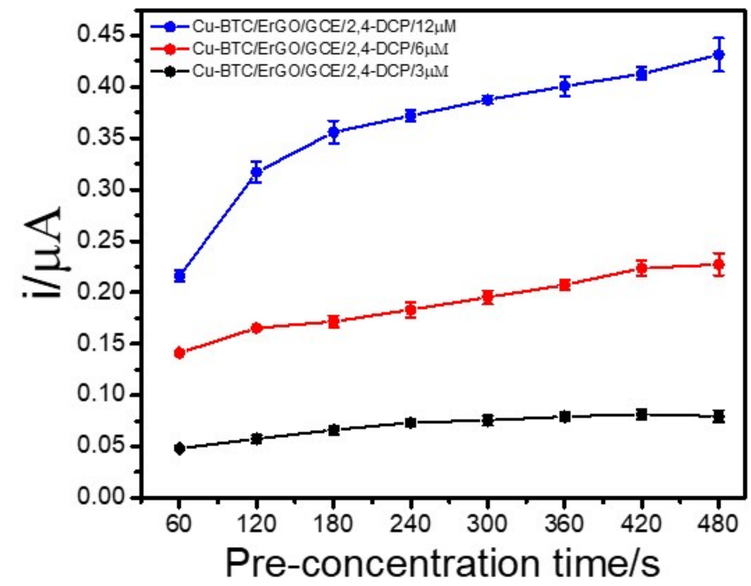

Figure 11S: Influence of accumulation time (60- 480 s) on DPV signals recorded in PBS solution (pH 7.0) containing of 2,4-DCP at concentration of 3 μM, 6 μM, 12μM on Cu-BTC/ErGO/GCE

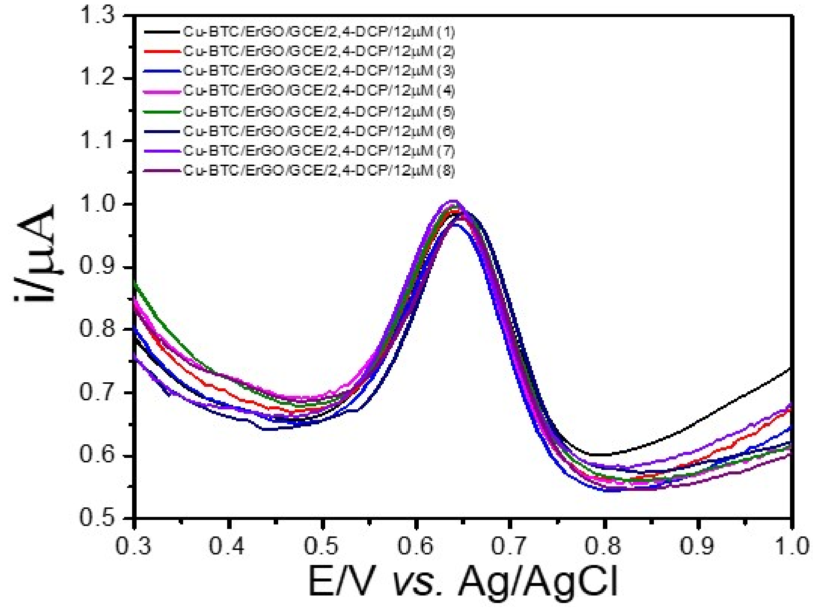

Figure 12S: Reproducibility of eight Cu-BTC/ErGO/GCE sensors at 12 μM 2,4-DCP in PBS pH 7

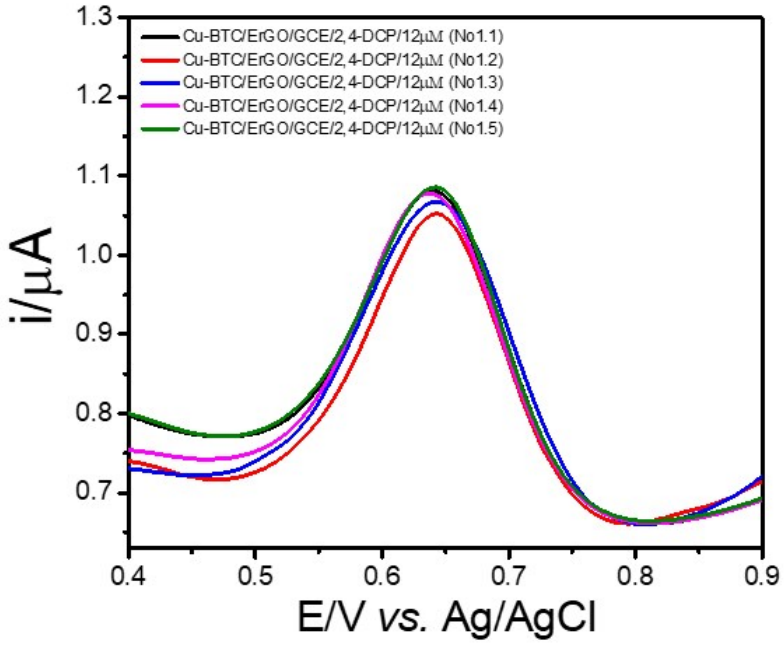

Figure 13S: Repeatability of 5 measurements using one Cu-BTC/ErGO/GCE sensor with 12 µM 2,4-DCP in PBS pH 7

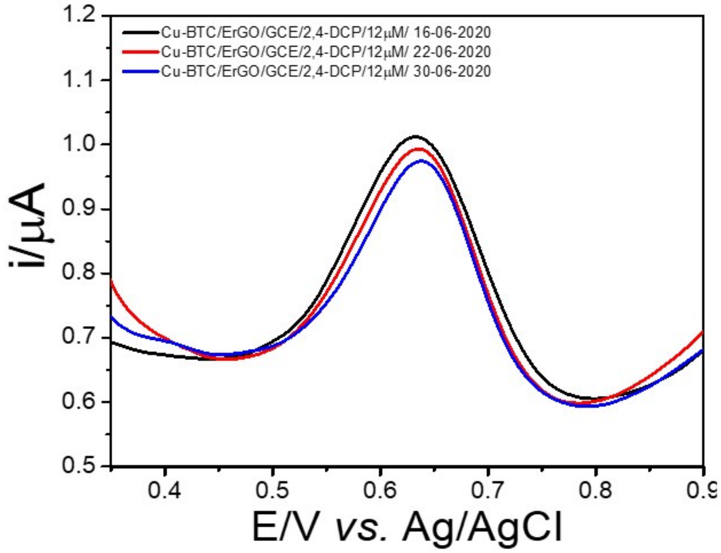

Figure 14S: DPASV response of Cu-BTC/ErGO/GCE for the detection of 2,4-DCP 12 µM after 2 weeks.

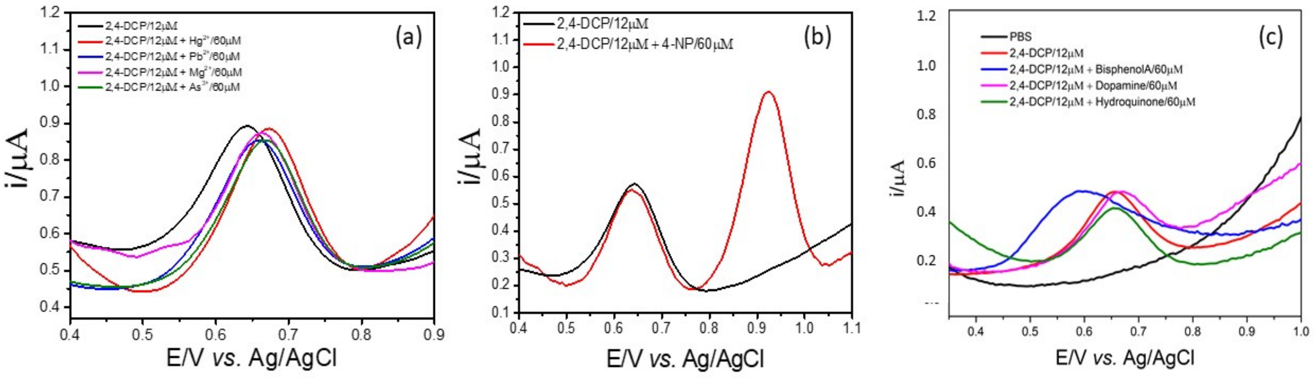

Figure 15S: Voltammograms of 2,4-DCP on Cu-BTC/ErGO/GCE before and after adding interferents at concentrations 5 time higher than that of analyte, 2,4-DCP with Hg^{2+}, Pb^{2+}, Mn^{2+} and As^{3+} (a) with 4-nitrophenol (b) and bisphenol A, hydroquinone, dopamine (c)

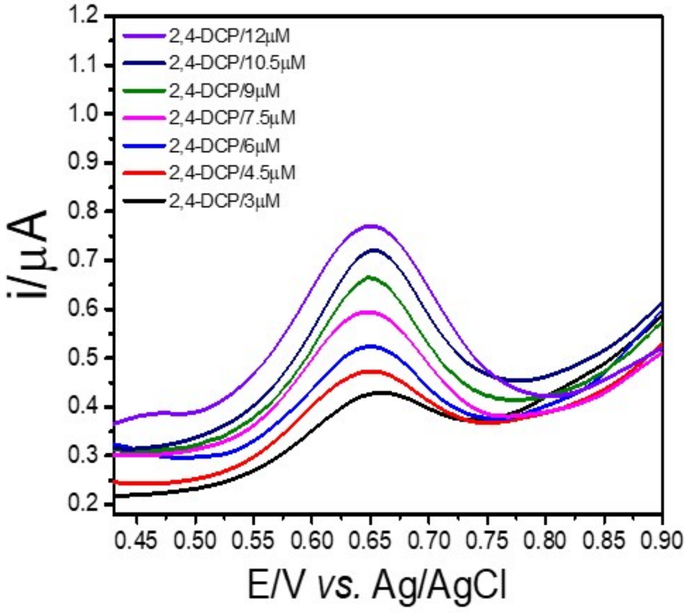

Figure 16S: Voltammograms of lake water spiked 2,4-DCP at different concentrations recorded on Cu-BTC/ErGO/GCE sensor
